# Physicochemical Characteristics and Flavor Properties of Texturized Dual-Proteins Extrudates: Effect of Surimi to Soybean Flour Ratio

**DOI:** 10.3390/foods11223640

**Published:** 2022-11-14

**Authors:** Xiaodong Li, Tonghao Zhang, Yueqi An, Tao Yin, Shanbai Xiong, Hongshan Rong

**Affiliations:** 1 College of Food Science and Technology, National R&D Branch Center for Conventional Freshwater Fish Processing (Wuhan), Huazhong Agricultural University, Wuhan 430070, China; 2 Engineering Research Center of Green Development for Conventional Aquatic Biological Industry in the Yangtze River Economic Belt, Ministry of Education, Wuhan 430070, China; 3 Tianmen Jifude Bean Products Co., Ltd., Tianmen 431700, China

**Keywords:** dual-proteins, extrudates, physicochemical characteristics, flavor properties

## Abstract

This study investigated the effects of surimi to soybean flour ratio (0:10, 1:9, 2:8, 3:7, 4:6) on the physicochemical characteristics and flavor properties of dual-proteins extrudates. The increasing ratio of surimi improved the color of extrudates and raised the apparent viscosity of the mixed raw materials, which led to the decrease of extrudates’ thickness. The excess ratio of surimi and soybean flour (more than 2:8) was bad for extrudates’ physicochemical characteristics with sharply decreased tensile strength, macroscopic longitudinal fracture, broken and unevenly distributed microstructure, increased water mobility and decreased free water content. However, the increasing ratio of surimi had no effect on the protein secondary structure of extrudates. Sensory evaluation, E—tongue and E—nose analysis suggested that adding surimi significantly changed the flavor properties of extrudates, with increased sweetness and umami taste, and an appropriate ratio (2:8 or 3:7) could reduce the beany flavor and without an obvious fishy off-flavor.

## 1. Introduction

Studies have shown that mixed protein foods (containing both plant and animal proteins) possess multiple health benefits due to their balanced nutritional composition and high bioavailability. Moreover, mixed protein foods meet the requirement of ecologically sustainable development [1]. Therefore, mixed protein foods have attracted much attention and inspired a research upsurge based on those advantages all over the world. Developed countries play a leading role in the research and development of new mixed protein foods [1]. In China, the development of mixed protein foods is defined as a dual-protein project which have been written into the guidelines of the National Nutrition Plan (2017–2030) [2].

The processing techniques of mixed protein foods include some traditional techniques such as thermal processing, fermentation, chemical-based protein coagulation, high-pressure homogenization, ultrasound, etc., and some new processing techniques such as 3D printing and extrusion technology [3]. Extrusion technology is where raw materials are pushed forward by a screw, affected by the joint action of high temperature, high pressure and shear force, and forming samples with specific shapes after passing through specific abrasives [4]. Compared with other methods of processing mixed protein food, extrusion technology requires less on the functional characteristics of raw protein. At the same time, extrusion technology has the advantages of simple processing, short-term high temperature, low nutritional loss and continuous large-scale production, making it suitable for the processing of mixed protein food [5]. However, at present, extrusion technology mainly uses grain starch and plant protein as raw materials to produce texturized products, while animal or fish protein is rarely used in extrusion. In recent years, researchers have tried to produce mixed-protein extruded food [6].

Fish is a potential protein source for producing texturized extrudates due to its high availability, high nutritional value and lack of connected religious problems [7]. To date, few researchers have used fish protein as a raw material to produce extruded food. Fish protein in the form of dried fish protein powder [8], leftover from aquatic processing [9] and commercial frozen surimi [10] were mixed with plant proteins or starch to prepare extrudates with low mixture and a porous structure by a single-screw extruder [11], or extrudates with high moisture and compact fiber structure by a twin-screw extruder [9,10]. Specially, chum salmon fish paste, including skin and bones [12], and the fish paste from whole or gutted Baltic Herring [13] were used to prepare dual-protein extrudates, which improved the utilization and avoided the complex pretreatment of fish. In terms of process conditions, high and low moisture extrusion technology were used to prepare extrudates with fish protein. Previous literature indicated that moisture content and temperature had significant effects on the quality of extrudates [9,11]. In general, the existing studies on the co-extrusion of fish and plant protein mainly focus on the optimization of processing parameters and material formula. Textural characteristics, structure and flavor are vital characteristics of texturized products. However, there are few reports regarding the mechanism of the formation of texture and structure of dual-proteins extruded products containing fish protein. Furthermore, the existing studies on the flavor properties of extrudates focus on reducing or eliminating the off-flavor from the plant proteins and simulating the meat flavor [14]. The flavor properties of extrudates with fish and plant proteins have rarely been studied.

Soybean proteins are widely used as raw material to produce texturized extrudates due to their high availability, low price, abundant essential amino acids and excellent ability to cohesion and texturize [15]. Therefore, in this study, frozen surimi (predominately fish protein) and soybean flour were used as raw materials to prepare dual-protein texturized extrudates by a single-screw extruder. The physicochemical characteristics of the extrudates were analyzed. Furthermore, the mechanism of surimi to soybean flour ratio affecting the physicochemical characteristics of extrudates was investigated by analyzing rheological properties, microstructure, water distribution and protein secondary structure. At last, the flavor properties of extrudates were measured by sensory evaluation, electronic tongue (E—tongue) and electronic nose (E—nose) analyzers.

## 2. Materials and Methods

### 2.1. Materials

Frozen silver carp (*Hypophthalmichthys molitrix*) surimi (75.6% moisture, 16.5% protein, 0.5% fat) was purchased from Jingli Aquatic Food Co., Ltd. (Honghu, Hubei, China). Soybean flour (10.6% moisture, 39.8% protein, 7.1% fat) was purchased from the Specialized Cooperative of Chengdou Bean-products (Zaozhuang, Shandong, China).

### 2.2. Rheological Properties of Raw Mixtures

Rheological properties of mixed raw materials were determined according to the method of Chen et al. [16]. Surimi and soybean flour were mixed under different mass ratios (*w*/*w*): 0:10, 1:9, 2:8, 3:7, 4:6. After adjusting the moisture to 80%, the mixtures were mixed well and stored at 4 °C overnight for the purpose of complete hydration. A steady shear test was carried out at the shear rate range of 1–100 s^−1^ on a rheometer (Discovery HR-2, TA Instruments, New Castle, DE, USA) with aluminum parallel plate geometry (40 mm diameter, 1 mm gap). The mixtures were loaded on the rheometer plate at 25 °C. A thin layer of low viscosity paraffin oil was added on the edge to prevent evaporation during measurement. The apparent viscosity, shear rate and shear stress of mixtures were recorded. Flow behavior is depicted by the power law model:

(1) $$\tau ={K\gamma }^{n}$$

where τ is the shear stress (mPa), K the consistency coefficient, γ the shear rate (s^−1^), and n the flow behavior index.

The frequency sweep test was carried out with the increasing angular frequency range from 0.1 to100 rad/s at the linear viscoelastic region.

### 2.3. Preparation of Texturized Extrudates

Frozen surimi was broken after moderate thawing. Surimi and soybean flour were configured according to mass ratio (*w*/*w*): 0:10, 1:9, 2:8, 3:7, 4:6. An appropriate amount of water was added to adjust the moisture of the mixtures to 37%. Then, the mixtures were thoroughly mixed in a mixer (JHF-20L, Zhengzhou Jinhe Machinery Manufacture Co., Ltd., Zhengzhou, Henan, China) for 20 min and stood for 30 min. Extrusion were conducted with a single-screw extruder (YYFS-90, Shandong Yuya Soybean Machinery Manufacturing Co., Ltd., Jinan, Shandong, China) with a screw length to diameter ratio of 25:1 and four heating zones. The temperature of each zone was set as 70 °C, 120 °C, 240 °C and 250 °C, respectively, and the screw speed 28 Hz. The extrudates were collected and cooled down to room temperature, then sealed in plastic bags and stored at −20 °C for the following tests.

### 2.4. Color and Thickness

The color of extrudates was determined by a hand−hold colorimeter (CR-400, Konica Minolta, Tokyo, Japan) in triplicate. The colorimeter was calibrated with a white plate and the values of lightness (L*), redness (a*) and yellowness (b*) were obtained. The value of whiteness (W*) was calculated by the following equation:

(2) $$W^{*}=100-\sqrt{\left(100-L^{*}\right)\times \left(100-L^{*}\right)+a^{*}\times a^{*}+b^{*}\times b^{*}}$$

The thickness of extrudates was measured by a spiral micrometer (DL9325, Deli Group Co., Ltd., Ningbo, Zhejiang, China).

### 2.5. Tensile Properties

The tensile properties of extrudates were measured by a Texture Analyzer (TA-XT plus, Stable Micro System, Godalming, Surrey, UK) according to the method of Zhang et al. [17]. A rectangular sample (30 mm × 90 mm) was obtained from fresh extrudates and was stretched using an A/TG probe at a speed of 1 mm s^−1^ until the strip was broken, at which the tensile resistant force was recorded.

### 2.6. Microstructure

The fresh extrudates were sliced and mounted by glutaraldehyde for 48 h, followed by dehydration with a graded series of ethanol solutions and critical point drying with CO_2_. Then the samples were placed on a bronze stub and sprayed with gold for 5 min. The images were taken with a scanning electron microscopy (SEM) (JSM-6390/LV, JEOL Ltd., Tokyo, Japan) at magnifications of 300× and 2000×.

### 2.7. Low−Field Nuclear Magnetic (LF−NMR) Resonance Measurements

The water status of the extrudates were characterized with an NMR analyzer (MR25, Niumag Electronics Technology Co., Ltd., Shanghai, China) at 32 °C according to the method of Li et al. [18]. The transverse relaxation time (T_2_) was measured with the Carr–Purcell–Meiboom–Gill (CPMG) sequence. The relaxation times (T_2b_, T_21_ and T_22_) and their corresponding relaxation signal component (A_2b_, A_21_ and A_22_) were recorded.

### 2.8. Fourier Transform Infrared Spectroscopy (FT−IR) Determination

The freeze-dried samples of extrudates were mixed with KBr and compressed to pellets before being analyzed with a FT-IR spectrometer (Nicolet iS10, Thermo Fisher Scientific, Waltham, MA, USA) at room temperature. The FT-IR spectra were recorded in the range of 400–4000 cm^−1^ with 32 scans at 4 cm^−1^ resolution. Peak-fit software (Version 4.12, SPSS, Chicago, IL, USA) was used to analyze the secondary structure of the proteins.

### 2.9. Sensory Evaluation of Flavor

Sensory evaluation of flavor (taste and odor) properties were conducted by 8 trained panelists with professional food backgrounds; the sensory scoring standard is shown in Table S1. The taste evaluation of extrudates was carried out at room temperature without further treatment. For odor evaluation, 4.00 g crushed extrudate was put into a 20 mL headspace bottle and heated in water bath at 50 °C.

### 2.10. E—Tongue Analysis

The analysis of the taste profile of extrudates was performed using an E—tongue analyzer (ASTREE II, Alpha MOS, Toulouse, Haute-Garonne, France) with 7 detection sensors. Briefly, the taste components were extracted by combining crushed extrudates (10.00 g) and 80 mL high purity water and shaking for 30 min. Then, the mixtures were centrifuged for 10 min at 11 kg and 4 °C, followed by diluting to 100 mL for E-tongue analysis. The sensors collected data once per second and the total acquisition time was 120 s. The response value of each sensor at 120 s was selected for analysis [19].

### 2.11. E—Nose Analysis

The aroma profile of extrudates was performed using an E—nose analyzer (Fox 4000, Alpha MOS, Toulouse, Haute-Garonne, France) with 17 metal oxide gas sensors. Approximately 2.00 g of the crushed extrudates were put into 20 mL headspace bottle. The bottles were incubated at 50 °C for 5 min. Then the injection needle absorbed 2.5 mL headspace gas at 60 °C and injected into the machine. The acquisition time was 120 s and the E-nose system was cleaning with clean gas for 1080 s after each sample test [19].

### 2.12. Statistical Analysis

All data were performed and plotted using Origin 2018 software (Origin-Lab, Northampton, MA, USA) and presented as means ± standard deviations. Significance was determined using one-way ANOVA with SAS 9.2 program software (SAS Institute Inc., Cary, NC, USA) and followed by Duncan’s test at *p* < 0.05.

## 3. Results and Discussion

### 3.1. Rheological Properties of Raw Materials

**Figure 1:**
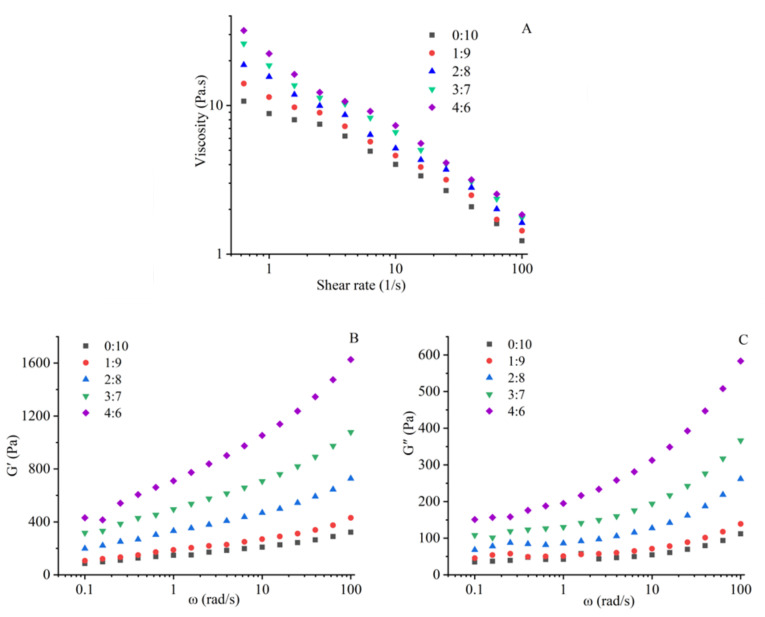
Rheological properties of the mixed raw materials with different surimi to soybean flour ratio. (**A**) the relationship of shear stress and apparent viscosity; (**B**) variation of storage modulus (G′); (**C**) variation of loss modulus (G″).

As shown in Figure 1A, the apparent viscosity of samples decreased with the increase of shear rate, showing a shear-thinning phenomenon, which was due to the destruction of intramolecular or intermolecular interactions [16,20]. Flow behavior is depicted by the power law model and the fitting results of the flow curve are presented in Table S2. The n values were all less than 1, which indicated that the mixed raw materials of surimi and soybean flour were pseudoplastic fluids [16]. Figure 1A and Table S2 show that the apparent viscosity increased significantly with the increasing ratio of surimi. Correspondingly, the K value increased from 13.75 to 25.50, while the n value decreased from 0.50 to 0.43, indicating reduced fluidity. As shown in Figure 1B,C, the storage modulus (G′) and loss modulus (G″) of samples increased gradually with the increasing angular frequency, which might be due to the formation of temporary cross-linking of protein molecular chains during the oscillation. Additionally, the G′ values of all samples were consistently higher than G″, meaning more solid-like behavior dispersions [16,20]. The increasing ratio of surimi both increased the values of G′ and G″ of mixed raw materials, which might be related to the fact that the viscosity of surimi was higher than soybean flour [21]. Additionally, due to the extremely low content of fat in surimi and approximately 7.1% lipid in soybean flour, the increasing ratio of surimi reduced the content of lipid (for lubrication) in the mixture, which might also lead to the increase of the viscosity, G′ and G″.

Under the same extrusion conditions, the increasing viscosity of the mixed raw materials would enhance the friction among the material, screw and barrel, resulting in a longer residence time of the material in the extruder and a higher pressure in the extruder cavity [22]. The residence time and pressure would affect the association, aggregation and re-aggregation of the protein and the interactions between protein and non-protein components, which would determine the apparent morphology and structural properties of the extrudates [23].

### 3.2. Appearances, Color and Thickness

**Figure 2:**
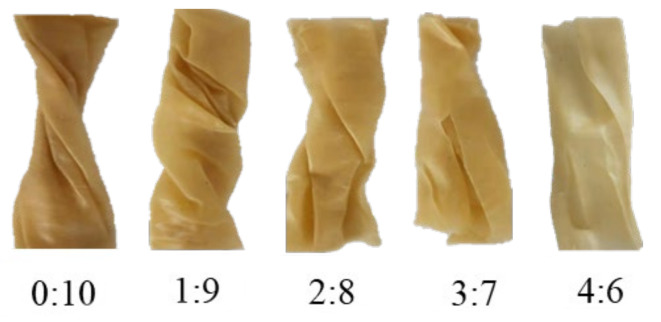
Appearances images of extrudates with different surimi to soybean flour ratio.

The appearances and color of food are very important factors which directly affect consumers’ acceptance and choice. Figure 2 shows the appearances of extrudates with different surimi to soybean flour ratios. The extrudate was in the shape of cylinder when the materials had just come out of the extruder, and then rotated into a spiral shape with the rotation of the screw. When the ratio of surimi and soybean flour increased from 0:10 to 2:8, the extrudates all kept a complete spiral shape. However, it was found that the extrudates occurred longitudinal fractures along the extrusion direction and could not maintain the complete cylinder and spiral shapes when the ratio of surimi and soybean flour were greater than 2:8.

Grabowska et al. reported that the phase separation caused by thermodynamic incompatibility between protein and non-protein components under the molten state was the prerequisite for the formation of stable anisotropic structure during extrusion [24]. The soybean flour used in this research consisted of abundant non-protein components (fiber, oil, etc.), while the non-protein components in surimi were relatively less. When the ratio of surimi and soybean flour was no more than 2:8, the increase of surimi did not significantly reduce the content of non-protein components, which might slightly decrease the degree of phase separation. Therefore, the extrudates could still form a stable anisotropic fiber structure. However, when the ratio of surimi and soybean flour was more than 2:8, the content of non-protein components reduced sharply, which might intensely weaken the degree of phase separation and reduce the ability to form the stable anisotropic structure. Furthermore, the microstructure of soybean protein might be disrupted due to the formation of excess independent surimi gel. Consequently, the extrudates experienced longitudinal fracture.

The color of extrudates mainly depends on the color of raw materials and the Maillard reaction during extrusion. It is clearly visible from Figure 2 that the color of the extrudates changed obviously with the increasing proportion of surimi: the yellow faded and the brightness increased gradually. Correspondingly, as shown in Table 1, with the ratio increasing from 0:10 to 4:6, the L* and W* values of the extrudates significantly increased by 6.02% and 10.75%, respectively, but the b* value significantly decreased by 10.23%. The color change was mainly attributed to the decreased proportion of the yellow soybean flour and the increased proportion of the grayish white frozen surimi. Meanwhile, the extrudates gradually became thinner (Table 1), which increased the transmittance of light and made the extrudates become brighter.

As shown in Table 1, the thickness of the extrudates obviously decreased from 0.415 mm to 0.325 mm when the surimi to soybean flour ratio increased from 0:10 to 4:6, which was mainly due to the higher extrusion pressure and the longer residence time caused by the increased viscosity of mixed raw materials (Figure 1A).

### 3.3. Tensile Properties

**Figure 3:**
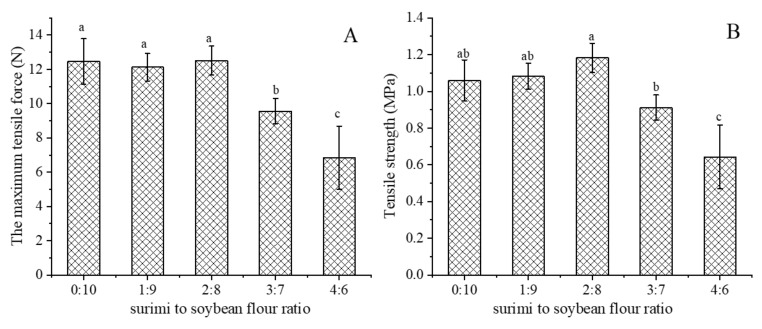
The maximum tensile force (**A**) and tensile strength (**B**) of extrudates with different surimi to soybean flour ratio. Different letters mean significant differences (*p* < 0.05).

Figure 3A,B show the tensile properties of extrudates with different surimi to soybean flour ratios. When the ratio of surimi and soybean flour increased from 0:10 to 2:8, the maximum tensile force of the extrudates did not change significantly. However, the maximum tensile force decreased sharply (*p* < 0.05) when the ratio was more than 2:8. The decrease in tensile force was mainly related to the decrease of thickness (Table 1) and tensile strength of extrudates.

The tensile strength of extrudates increased slightly at first (*p* > 0.05) when the ratio of surimi and soybean flour increased from 0:10 to 2:8. This might be due to the fact that the surimi formed a small independent gel, which might filled into the voids of the matrix of extrudates [25]. However, when the ratio was more than 2:8, the tensile strength decreased sharply (*p* < 0.05). The main reason might be that the excess surimi significantly weakened the degree of phase separation, and therefore the extrudates formed an unstable anisotropic fiber structure. Previous research reported that the mixed animal and plant proteins with distinct gelation mechanisms would form an independent gel structure after thermal treatment [26]. Additionally, there might be no strong interactions between the independent gel which had a negative effect on the gel strength [26]. Therefore, besides the phase separation, the formation of independent gel structure might lead to the decrease of the tensile strength as well.

### 3.4. Microstructure

**Figure 4:**
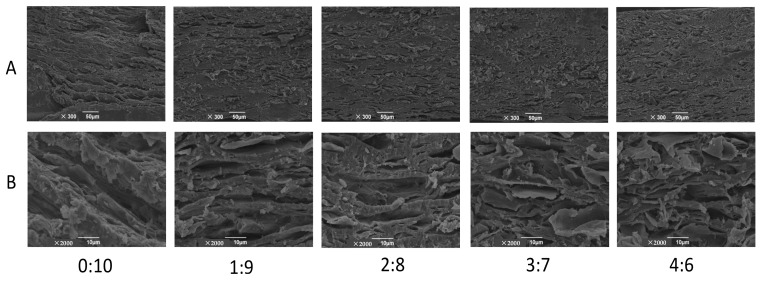
SEM images of extrudates with different surimi to soybean flour ratios ((**A**)-300×; (**B**)-2000×).

As shown in Figure 4, the microstructure of extrudate without surimi showed a bunchy fiber structure arranged along the extrusion direction. When the ratio of surimi and soybean flour were 1:9 and 2:8, the microstructure of the extrudates gradually became more compact, although the bunchy fiber structure still remained unbroken. However, when the surimi to soybean flour ratio were 3:7 and 4:6, plenty of broken and uneven flakes were observed among the bunchy fibers, which indicated that the excess proportion of surimi was not conducive to the microstructure of extrudates.

Adding a small amount of surimi might have little effect on the degree of phase separation during extrusion, and therefore the extrudates still formed a relatively stable anisotropic fiber structure [24]. On the other side, this part of surimi might form independent gel and adhered to the voids of matrix of soybean protein gel, resulting in less effect to its microstructure. However, the excess proportion of surimi might seriously weaken the degree of phase separation and the extrudates could not form a stable anisotropic fiber structure [24]. Furthermore, the aggregation of an independent gel of surimi decreased the continuity of the microstructure of the soybean protein gel [26]. Additionally, the rising pressure of the extruder cavity caused by the increasing surimi to soybean flour ratio might also have led to the formation of compact microstructure and flakes. The deteriorated microstructure further explained the fracture and the significant decrease of tensile properties of the extruded samples at high surimi to soybean flour ratio (Figure 2 and Figure 3).

### 3.5. Analysis of Water Status

LF-NMR technology is widely used to measure T_2_ relaxation time, which can characterize the status and distribution of water in food. T_2_ relaxation time curve includes three peaks with the increasing degree of water freedom: T_2b_, T_21_ and T_22_, which corresponded to bound water, immobilized water and free water [27]. The T_2_ relaxation time and water fractions of extrudates with different surimi to soybean flour ratio are shown in Table 2. Three relaxation time (T_2b_, T_21_ and T_22_) and corresponding proportions (A_2B_, A_21_ and A_22_) were determined for all extrudates. The proportion of A_21_ was the highest (more than 83%), which indicated the exiting status of water for the extrudates was mainly immobilized water [25].

A previous study reported that T_2b_, mainly contained non-exchangeable CH protons, represented the most stable part of water molecules [28]. As shown in Table 2, T_2b_ increased while A_2b_ decreased gradually with the increasing ratio of surimi to soybean flour, which meant that adding surimi decreased the non-exchangeable CH protons in the extrudates and increased the degree of freedom of bound water. The reason for this was that the increasing proportion of surimi decreased the content of non-protein components such as dietary fiber with abundant hydrophilic groups, which could bound water molecules closely [29]. In addition, the high temperature (250 °C), higher shear force and pressure caused by the increase of viscosity might seriously disrupt the native structure of macromolecules and reduce their ability to bind with water tightly. The results showed that the T_21_ increased which indicated that adding surimi increased the degree of freedom of immobilized water. The immobilized water was hydrated with macromolecules and distributed in the internal voids of extrudates [25]. The increasing ratio of surimi and soybean flour disrupted the integrity of the extrudate microstructure and created more voids, which eventually led to an upward trend of A_21_. As compared to the sample without surimi, T_22_ was obviously lower when the surimi to soybean flour ratio were 3:7 and 4:6. The main reason for this was that the extrudates occurred fractures under these additions, increasing the mobility of free water. The water evaporated due to a sudden drop in pressure when the materials had just come out of the extruder. The amount of the evaporated water depends on the structure of the extrudates. The integrity of the extrudates’ structure was severely disrupted under high surimi ratios, which might evaporate high amounts of free water and decrease the content of A_22_. Consequently, the relative proportion of A_21_ increased [30].

### 3.6. Analysis of Protein Secondary Structure by FT-IR

The amide I band (1700–1600 cm^−1^), which contained approximately 80% information of the C=O stretching vibration in the protein infrared spectrum, was used to detect the secondary structure of proteins. The amide I band is composed of several overlapping protein secondary structures, including α-helix, β-sheet, antiparallel β1 and β2 sheet, β-turns and random coils [31]. The positions of the second-derivative band of extrudates (Figure S1A–G and Table 3) are consistent with the previous literature which reported that the antiparallel β1-sheet was located at 1608–1622 cm^−1^; the β-sheet was 1622–1637 cm^−1^; the random coil was 1637–1645 cm^−1^; the α-helix band was 1646–1662 cm^−1^; the β-turn was 1662–1681 cm^−1^ and the antiparallel β2-sheet was 1682–1700 cm^−1^ [32].

For raw materials, Table 3 shows that the main protein secondary structure of soybean was β-sheet (including antiparallel β1and β2 sheet), which was consistent with the previous research that soybean protein was rich in globulins with a β-sheet structure [31]. A previous study showed that the main protein secondary structure of fresh surimi was α-helix [33]. However, the main protein secondary structure of raw surimi in our study was β-sheet. The main reason for this is that the raw frozen silver carp surimi we used had been stored (frozen) for half a year, the tail structure of myosin would change obviously with the extension of frozen storage time, leading to the decrease of α-helix content [34].

Table 3 shows that the β1-sheet and β-sheet of extrudate without surimi markedly increased by 41.67% and 7.5% compared with raw soybean protein, while the random coil, α-helix, β-turn and β2-sheet decreased by 3.35%, 7.34%, 8.70% and 10.64% respectively. The high temperature, high pressure and high shear force during the extrusion promoted the unfolding of the soybean protein molecular chain, which caused the exposure of the internal hydrophobic regions and hydrogen bonds, and the increase of β1-sheet and β-sheet [35].

There were no significant differences in the secondary structure of the extrudates when the ratios of surimi and soybean flour were 1:9, 2:8, 3:7 and 4:6, which meant that intermolecular and intramolecular hydrogen bonds of extrudates were relatively stable and the surimi to soybean flour ratio had no effect on the protein secondary structure of the extrudates [36]. It might be that fish protein and soybean protein formed an independent gel structure during extrusion, and there was no strong interaction between the two gels [26].

### 3.7. Mechanism Explanation on the Structural Properties of Extrudates

**Figure 5:**
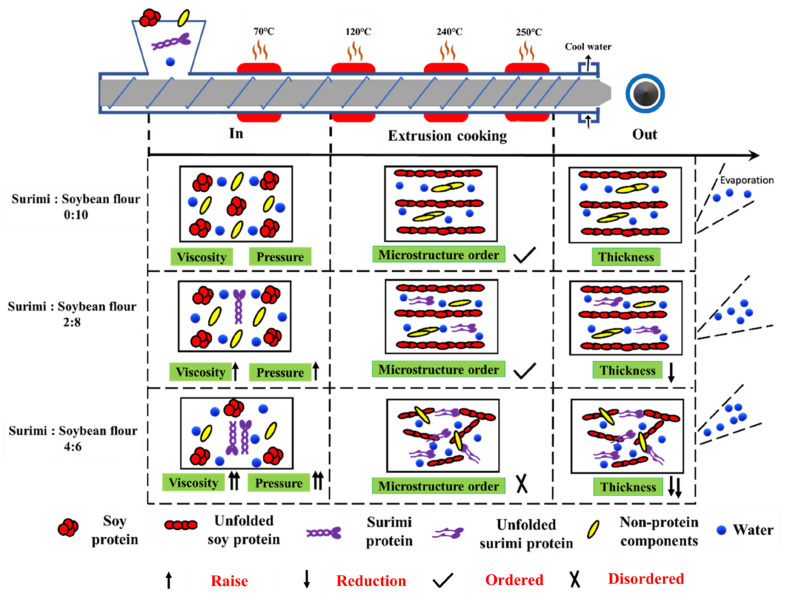
The mechanism on structure properties of dual-protein extrudates as affected by the ratio of surimi and soybean flour.

Based on the present results, the mechanism of the ratio of surimi and soybean flour affecting the structure properties of soybean-based extrudates is illustrated in Figure 5. When the ratio of surimi and soybean flour was 0:10, the even mixtures in the feeding port entered inside the extruder with the rotation of screw. The protein and non-protein components in the soybean flour experienced phase separation during the extrusion cooking process. Meanwhile, the protein molecular chain unfolded and then re-aggregated along the extrusion direction to form a stable anisotropic fiber structure (Figure 4). When the extrudate left the extrusion head, due to the sudden drop of pressure, part of the water evaporated, and finally a texturized product with fiber structure was formed.

The increasing proportion of surimi raised the viscosity of the mixture (Figure 1A and Table S2), resulting in a higher pressure in the extruder cavity which eventually decreased the thickness of extrudates (Table 1). When the ratio of surimi and soybean flour did not exceed 2:8, the degree of phase separation might have been hardly affected and the surimi gel filled into the voids of the soybean protein gel matrix. In addition, the increase of pressure caused by the viscosity made the microstructure of extrudates more compact (Figure 4). However, when the ratio of surimi and soybean flour was more than 2:8, the degree of phase separation might have been largely weakened due to the decrement of the non-protein components. Meanwhile, the increased aggregation of surimi decreased the continuity of soybean microstructure. These two factors eventually led to the longitudinal fracture (Figure 2) in the macrostructure of the extrudates, the sharply decreased tensile properties (Figure 3) and the disorder and the fragmentation in microstructure (Figure 4). On the hand, the disorder and fracture structure of the extrudates increased the degree of water freedom and decreased the relative proportion of free water as more water was evaporated (Table 2).

### 3.8. Sensory Evaluation of Flavor

It can be seen from Table 4 that the taste sensory scores of extrudates increased significantly with the increasing ratio of surimi, meaning that adding surimi improved the taste property of extrudates. In addition, sensory evaluation panelists declared that the positive taste of fish meat was mainly recognized in the aftertaste (after chewing the extrudates) and they declared that the increase of the sweet and umami taste contributed to the high sensory scores of extrudates, which might be because of the existence of sucrose (as antifreeze) and the rich umami components in frozen surimi [18].

Similarly, the odor sensory scores of extrudates without surimi were the lowest, which was due to the obvious beany flavor from the fat oxidation of soybean flour [14]. Then, the odor sensory scores of extrudates gradually increased when the ratio of surimi and soybean flour was 1:9 and 2:8, however, the excess ratio (3:7 and 4:6) led to a slight decrease of sensory scores. The reason for this was that the pleasant fish and sweet aroma caused by an appropriate ratio of surimi after extrusion might mask the beany flavor, but the excess ratio of surimi might lead to the emergence of a fishy off-flavor.

Overall, it was suggested that an appropriate ratio (2:8 or 3:7) of surimi might both improve the taste property with an increased sweet and umami taste, and reduce the beany flavor of extrudates and without an obvious fishy off-flavor.

### 3.9. E—Tongue Analysis

**Figure 6:**
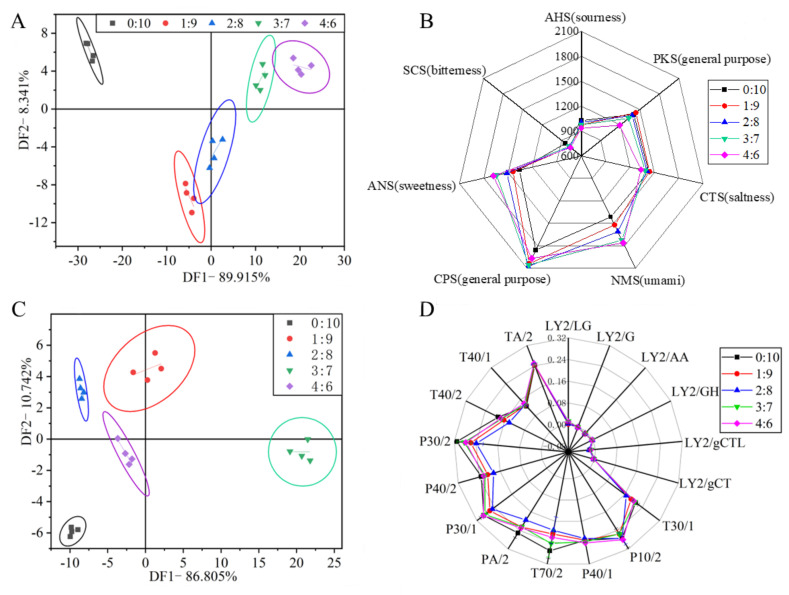
Flavor profiles of extrudates with different surimi to soybean flour ratio. DFA diagram (**A**) and radar graph (**B**) of E—tongue data; DFA diagram (**C**) and radar graph (**D**) of E—nose data.

The data collected by the E—tongue were statistically analyzed by discriminant factor analysis (DFA) and the result is shown in Figure 6A. The contribution rates of DF1 and DF2 were 89.915% and 8.341%, respectively, and the cumulative contribution rate reached 98.256% (greater than 85%), indicating that DFA basically reflected the overall taste profile of the extrudates [19]. In the DFA diagram, the spatial distance of different color areas indicated the taste distribution and the difference between sample groups. The taste profile of extrudate without surimi was in a quadrant alone and far away from the taste profile of extrudates with surimi. Additionally, with the increase of surimi ratio, the taste profile of the four groups of extrudates with surimi were regularly distributed along the increasing direction of DF1, and showed only a little overlap, demonstrating that adding surimi significantly changed the taste of extrudates.

A radar graph (Figure 6B) further confirmed the conclusion from the DFA analysis. It can be seen that the difference in taste profile of extrudates was mainly reflected by the changes of the umami taste (NMS values) and sweetness (ANS values). With the increase of the surimi ratio, the umami taste and sweetness response values of extrudates gradually increased, while the changes of sourness (AHS values), saltiness (CTS values) and bitterness (SCS values) were relatively small, which were consistent with the result of the taste sensory evaluation. The increase of sweetness was mainly attributed to the sucrose as an antifreeze in frozen surimi. In addition, the increase of umami taste was mainly attributed to the fact that frozen surimi contained umami substances such as free amino acids (aspartic acid and glutamic acid), nucleotides and carnosine [19].

### 3.10. E—Nose Analysis

Figure 6C shows the results of the DFA of the odor profile of extrudates with different surimi to soybean flour ratios according to the response values of E—nose. The contribution rates of DF1 and DF2 were 86.805% and 10.742%, respectively, and the cumulative contribution rate was 97.547% (greater than 85%), indicating that the DFA basically reflects the overall odor profile of the extrudates [19]. In the DFA diagram, the odor profile distribution of five sample groups are far away from each other and the fitting range was not crossed, indicating that the odor profile of extrudates occurred a significant change after adding different ratio of surimi.

As shown in radar graph (Figure 6D), the 6 L−series sensors had no response while the 6 P−series sensors and 5 T−series sensors had obvious responses. The odor profile of extrudates with surimi were almost inside the odor profile of extrudates without surimi, indicating that adding surimi might partly decrease the content of volatile odor components. Moreover, the odor profile of the extrudates with different surimi to soybean flour ratio were similar in the shape, but the response values of the partial sensors had a significant difference. The differences were mainly related to the response values of 7 sensors including P30/1, P30/2, P40/2, PA/2, T30/1, T40/2 and T70/2, and the response values of the 7 sensors exhibited a trend of decreasing first and then increasing with the increasing surimi ratio, indicating that the contents of aromatics, sulfides and aldehydes compounds changed significantly [37]. Previous studies have shown that the aldehydes were the main compounds of the beany flavor [38] and fishy off-flavor [39]. The decrease of the response value of P30/2 sensor first (sensitive to aldehydes) might indicate the reduction of beany flavor while the followed increase might mean the emergence of fishy off-flavor.

## 4. Conclusions

The ratio of surimi to soybean flour had a significant effect on the properties of dual-protein extrudates. Adding more surimi resulted in a thinner extrudate with higher lightness and whiteness in color. The tensile strength increased slightly when the ratio of surimi and soybean flour was no more than 2:8. However, it decreased sharply as the ratio was further increased. The high ratio of surimi (>2:8) led to the fracture of extrudates in macrostructure, the disruption of microstructure integrity and the increase of water mobility, while free water content decreased. The changes in the structural and physical properties of the dual-protein extrudates might be mainly related to the change of viscosity of the mixed raw materials, phase separation and the formation of independent surimi and soybean protein gels that were not strongly interacted. Additionally, sensory evaluation combined with E—tongue and E—nose analyzers effectively identified the differences of the flavor properties of extrudates, and the increasing ratio of surimi was beneficial to taste properties, with increased sweet and umami taste, but excess surimi could reduce the odor property. However, the research on the flavor properties of dual-protein extrudates in this paper only revealed the changes of the external laws. The intrinsic reasons for the increase of the umami taste, the decrease of the beany flavor and the increase of the fishy off-flavor need to be further revealed by methods of molecular sensory science.

## Figures and Tables

**Table 1:** Color parameters and thickness of extrudates with different surimi to soybean flour ratio.

Surimi to Soybean Flour Ratio	L*	a*	b*	W*	Thickness (mm)
0:10	75.40 ± 0.01 ^d^	1.53 ± 0.29 ^a^	39.36 ± 0.33 ^a^	53.56 ± 0.16 ^e^	0.415 ± 0.009 ^a^
1:9	77.45 ± 0.26 ^c^	−0.47 ± 0.22 ^b^	39.23 ± 0.27 ^a^	54.75 ± 0.11 ^d^	0.405 ± 0.017 ^a^
2:8	78.34 ± 0.16 ^b^	−1.31 ± 0.05 ^c^	38.01 ± 0.11 ^b^	56.24 ± 0.01 ^c^	0.375 ± 0.017 ^b^
3:7	80.26 ± 0.18 ^a^	−2.04 ± 0.13 ^d^	36.60 ± 0.09 ^c^	58.35 ± 0.02 ^b^	0.355 ± 0.009 ^b^
4:6	79.94 ± 0.16 ^a^	−1.95 ± 0.12 ^cd^	35.33 ± 0.50 ^d^	59.32 ± 0.52 ^a^	0.325 ± 0.009 ^c^

Note: Different letters in the same column mean significant differences (*p* < 0.05).

**Table 2:** Relaxation times and corresponding peak areas of extrudates with different surimi to soybean flour ratios.

Surimi to Soybean Flour Ratio	T_2_/(ms)			A_2_/(%)		
	T_2b_	T_21_	T_22_	A_2b_	A_21_	A_22_
0:10	0.26 ± 0.01 ^bc^	4.92 ± 0.01 ^c^	125.26 ± 2.17 ^ab^	9.78 ± 0.48 ^a^	83.30 ± 0.16 ^bc^	6.92 ± 0.08 ^a^
1:9	0.27 ± 0.02 ^b^	5.21 ± 0.06 ^b^	129.67 ± 2.24 ^ab^	9.22 ± 0.64 ^ab^	83.65 ± 0.51 ^b^	7.14 ± 0.04 ^a^
2:8	0.30 ± 0.01 ^ab^	5.74 ± 0.13 ^ab^	132.65 ± 0.73 ^a^	8.84 ± 0.17 ^ab^	84.26 ± 0.14 ^ab^	6.90 ± 0.17 ^ab^
3:7	0.32 ± 0.02 ^a^	6.33 ± 0.18 ^a^	130.92 ± 2.02 ^a^	8.42 ± 0.34 ^b^	84.53 ± 0.69 ^ab^	6.05 ± 0.07 ^c^
4:6	0.32 ± 0.01 ^a^	6.59 ± 0.12 ^a^	131.20 ± 0.72 ^a^	8.34 ± 0.11 ^b^	85.52 ± 0.28 ^a^	6.14 ± 0.21 ^c^

Note: Different letters in the same column mean significant differences (*p* < 0.05).

**Table 3:** The relative percentage of protein secondary structure for raw materials and extrudates with different surimi to soybean flour ratios.

Sample	Surimi to Soybean Flour Ratio	Secondary Structure/(%)					
		β1	β-Sheet	Random Coil	α-Helix	β-Turns	β2
Raw materials	Surimi	8.06 ± 0.10 ^c^	18.22 ± 0.03 ^bc^	23.46 ± 0.30 ^a^	23.22 ± 0.10 ^a^	16.57 ± 0.11 ^ab^	10.46 ± 0.16 ^ab^
	Soybean flour	8.76 ± 0.03 ^b^	18.62 ± 0.05 ^b^	22.10 ± 0.07 ^b^	21.93 ± 0.03 ^b^	17.13 ± 0.05 ^a^	11.46 ± 0.07 ^a^
Extrudates	0:10	12.41 ± 0.01 ^ab^	20.03 ± 0.11 ^a^	21.36 ± 0.21 ^c^	20.32 ± 0.15 ^c^	15.64 ± 0.07 ^bc^	10.24 ± 0.02 ^ab^
	1:9	13.33 ± 0.10 ^a^	20.54 ± 0.03 ^a^	21.65 ± 0.01 ^bc^	20.25 ± 0.15 ^c^	14.85 ± 0.14 ^c^	9.38 ± 0.13 ^bc^
	2:8	12.85 ± 0.17 ^a^	19.94 ± 0.21 ^a^	21.50 ± 0.25 ^bc^	20.37 ± 0.02 ^c^	15.45 ± 0.25 ^bc^	9.89 ± 0.39 ^b^
	3:7	12.81 ± 0.14 ^a^	20.13 ± 0.13 ^a^	21.59 ± 0.06 ^bc^	20.39 ± 0.02 ^c^	15.26 ± 0.16 ^bc^	9.83 ± 0.03 ^b^
	4:6	12.94 ± 0.08 ^a^	20.35 ± 0.13 ^a^	21.74 ± 0.11 ^bc^	20.37 ± 0.15 ^c^	15.12 ± 0.01 ^bc^	9.47 ± 0.17 ^bc^

Note: Different letters in the same column mean significant differences (*p* < 0.05).

**Table 4:** Sensory evaluation scores of flavor properties of extrudates with different surimi to soybean flour ratios.

Surimi to Soybean Flour Ratio	0:10	1:9	2:8	3:7	4:6
Taste	3.85 ± 0.76 ^cd^	4.68 ± 1.06 ^c^	5.70 ± 0.85 ^b^	6.14 ± 1.62 ^ab^	6.67 ± 1.14 ^a^
Odor	3.64 ± 1.21 ^c^	5.43 ± 1.34 ^b^	6.78 ± 1.12 ^a^	6.35 ± 1.25 ^ab^	5.82 ± 0.84 ^b^

Note: Different letters in the same line mean significant differences (*p* < 0.05).

## Data Availability

Data is contained within the article and the supplementary material.

## Supplementary Materials

The following supporting information can be downloaded at: https://www.mdpi.com/article/10.3390/foods11223640/s1, Table S1: Criteria for sensory evaluation; Table S2: Power law equation fitting results of steady shear flow curves of the raw materials with different surimi to soybean flour ratio; Figure S1: Fourier self-deconvoluted curve-fitted spectra (A-G) for raw materials and extrudates with different surimi to soybean flour ratio. A-raw frozen surimi; B-raw soybean flour; C-G extrudates with different addition percentages of surimi at 0:10, 1:9, 2:8, 3:7, 4:6, respectively.
