# Supplementary material for: Physicochemical Characteristics and Flavor Properties of Texturized Dual-Proteins Extrudates: Effect of Surimi to Soybean Flour Ratio
Source: Foods. 2022 Nov 14;11(22):3640. doi: 10.3390/foods11223640 (PMC9689315; doi:10.3390/foods11223640)
Supplement: Supplementary file 1 [file foods-11-03640-s001.zip › foods-1987927-supplementary.pdf]

Table S1. Criteria for sensory evaluation.

Project	Standard description	Score
Taste	Soybean taste, strong and delicious fish aftertaste, strong sweet and umami taste	8-10
	Soybean taste, obvious fish aftertaste, obvious sweet and umami taste, no bitter taste	6-8
	Soybean taste, slightly fish aftertaste, slightly sweet and umami taste, no bitter taste	4-6
	Strong soybean taste, no fish aftertaste, basically no sweet and umami taste, slightly bitter taste	2-4
	Strong soybean taste, no fish aftertaste, obvious bitter taste	0-2
Odor	Rich soybean and fish aroma, no beany and fishy off-flavor	8-10
	Obvious soybean and fish aroma, basically no beany and fishy off-flavor	6-8
	Obvious soybean aroma, slightly fish aroma, slightly beany or fishy off-flavor	4-6
	Obvious soybean aroma, no odor of fish, obvious beany or fishy off-flavor	2-4
	Slightly soybean aroma, no odor of fish, strong beany or fishy off-flavor	0-2

Table S2. Power law equation fitting results of steady shear flow curves of the raw materials with different surimi to soybean flour ratio.

Surimi to soybean flour ratio	K(10^{-3}Pa•s^n)	n	R^2
0:10	13.75 ± 0.97^{de}	0.50 ± 0.017^{a}	0.99137
1:9	14.79 ± 0.75^{d}	0.50 ± 0.013^{a}	0.99527
2:8	16.18 ± 0.64^{c}	0.49 ± 0.012^{a}	0.9953
3:7	22.45 ± 0.96^{b}	0.45 ± 0.011^{b}	0.99533
4:6	25.50 ± 1.17^{a}	0.43 ± 0.011^{bc}	0.99378

Note: Different letters in the same column mean significant differences (P < 0.05)

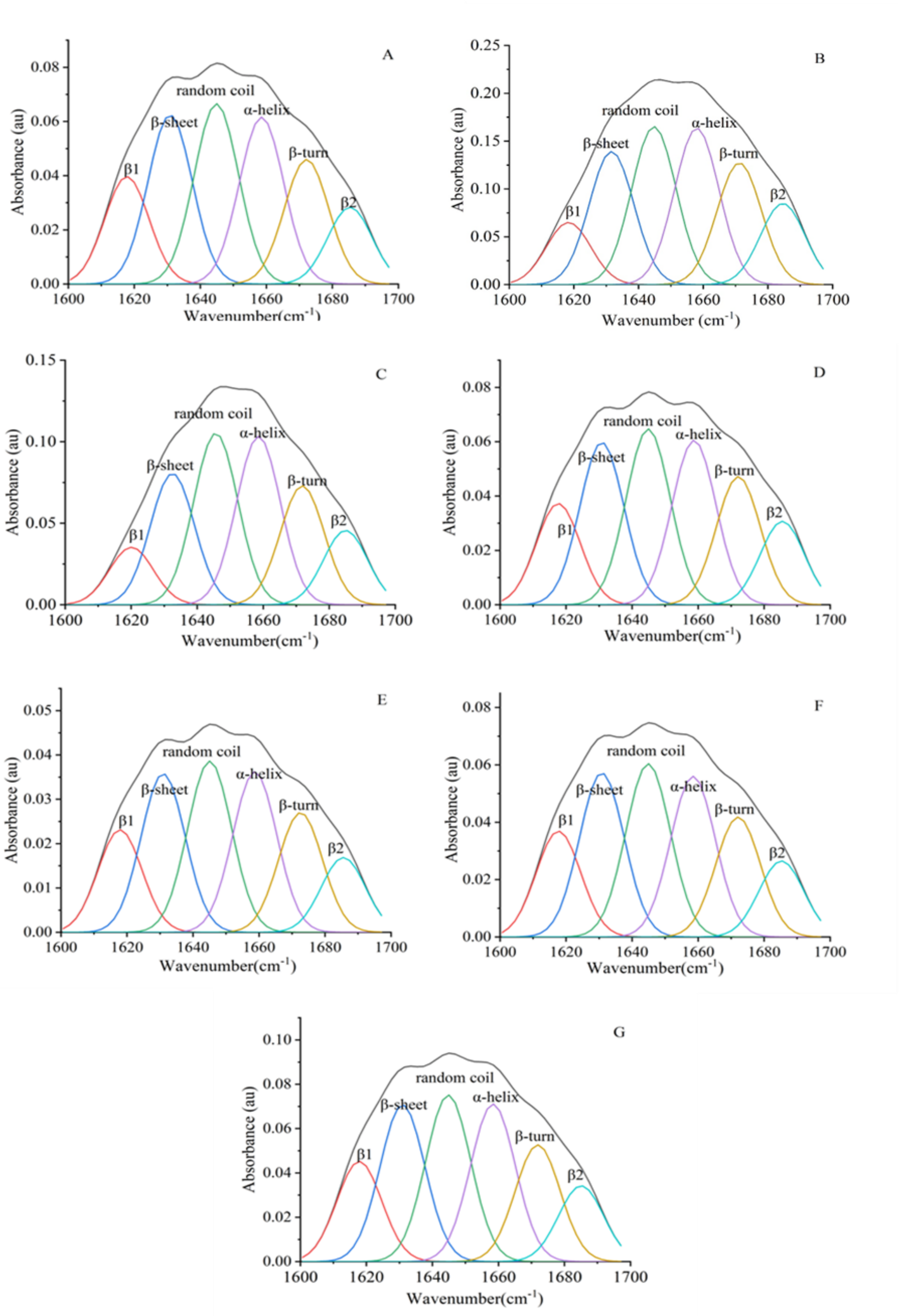

Figure S1. Fourier self-deconvoluted curve-fitted spectra (A-G) for raw materials and extrudates with different surimi to soybean flour ratio. A-raw frozen surimi; B-raw

soybean flour; C-G extrudates with different surimi to soybean flour ratio at 0:10, 1:9, 2:8, 3:7, 4:6, respectively.
